# Hydrogen sulphide donors selectively potentiate a green tea polyphenol EGCG-induced apoptosis of multiple myeloma cells

**DOI:** 10.1038/s41598-017-06879-5

**Published:** 2017-07-27

**Authors:** Jaehoon Bae, Motofumi Kumazoe, Shuya Yamashita, Hirofumi Tachibana

**Affiliations:** Division of Applied Biological Chemistry, Department of Bioscience and Biotechnology, Faculty of Agriculture, Kyushu University, Fukuoka, 812-8581 Japan

## Abstract

Hydrogen sulphide (H_2_S) is a colourless gas with the odour of rotten eggs and has recently been recognized as a signal mediator in physiological activities related with the regulation of homeostasis, the vascular system and the inflammatory system. Here we show that H_2_S donors, including sodium hydrogen sulphide (NaHS), GYY 4137 and diallyltrisulfide (DATS), synergistically enhanced the anti-cancer effect of a green tea polyphenol (−)-epigallocatechin-3-*O*-gallate (EGCG) against multiple myeloma cells without affecting normal cells. NaHS significantly potentiated the anti-cancer effect of EGCG and prolonged survival in a mouse xenograft model. In this mechanism, H_2_S enhanced apoptotic cell death through cyclic guanosine monophosphate (cGMP)/acid sphingomyelinase pathway induced by EGCG. Moreover, NaHS reduced the enzyme activity of cyclic nucleotide phosphodiesterase that is known as cGMP negative regulator. In conclusion, we identified H_2_S as a gasotransmitter that potentiates EGCG-induced cancer cell death.

## Introduction

Currently, there is extensive interest in the health benefits of green tea. An epidemiological study demonstrated that green tea consumption was associated with a low risk of hematologic malignancies^1^. The major constituent of green tea, (−)-epigallocatechin-3-*O*-gallate (EGCG), has been shown to have cancer-preventive and therapeutic effects^2–4^. We recently identified a 67-kDa laminin receptor (67LR) as the sensing molecule of EGCG^3, 4^. Interestingly, 67LR has been shown to be overexpressed in various types of cancers^4–8^. Indeed, EGCG selectively kills multiple myeloma (MM) cells by targeting cancer-overexpressed 67LR^4–7^. Moreover, EGCG induces apoptosis by upregulating cyclic guanosine monophosphate (cGMP) in cancer cells, including MM, acute myeloid leukaemia, pancreatic cancer and prostate cancer cells^4^. These results demonstrate the potent and specific anti-cancer activity of EGCG and provide the rationale for its clinical evaluation^7–9^. However, the plasma concentration of EGCG is not enough to eradicate cancer cells.

cGMP is well known as an intracellular second messenger that mediates a number of physiological processes including cardiovascular functions, neurotransmission and anti-cancer effect. cGMP is produced by soluble guanylate cyclase (sGC). sGC is the receptor for nitric oxide (NO) that is produced by nitric oxide synthase (NOS)^4, 10–15^. Cyclic nucleotide phosphodiesterases (PDEs) play a major role in cell signalling and are specifically negative regulators of cAMP and cGMP in mammalian tissues. cGMP-PDE is one of the enzymes of the PDE family and is a major inactivator of the intracellular messenger cGMP. Recently, it has been shown that specific PDE inhibitors might be new therapeutic approaches for numerous pathologies^16–18^. PDE5 is known to be a cGMP-specific enzyme that degrades cGMP^19^. PDE5 inhibitors have been shown to be effective for the treatment of erectile dysfunction^20^. Moreover, PDE5 inhibitor has been shown to increase cGMP-dependent apoptosis pathway in cancers^4, 5^. There are many recent studies on the discovery of new PDE5 inhibitors for the treatment of numerous pathologies.

Several studies have demonstrated that endogenous H_2_S acts as a gasotransmitter similar to NO and carbon monoxide in the human body^21^. In this study, we used several H_2_S donors, such as a NaHS, GYY 4137 and diallyltrisulfide (DATS). NaHS is an inorganic sulphide salt that has been widely used as H_2_S equivalents in many biological studies. NaHS rapidly releases H_2_S under laboratory conditions. On the other hand, GYY 4137 releases much lesser H_2_S and at a slower rate than NaHS but has shown sustained release of H_2_S in a culture medium. The release mechanism of H_2_S from NaHS and GYY 4127 are known to be by hydrolysis^22–24^. DATS is a garlic-derived sulphur compound that produces H_2_S. Release of H_2_S from sulphur compounds is facilitated by increasing the numbers of sulphur atoms and by allyl substituents. DATS, including trisulphides that undergo nucleophilic substitutions at the sulphur atom, then produces H_2_S. The mechanism of release of H_2_S from DATS is by thiol activation in which sulphur atoms react with cell membrane thiol^25^.

There are several recent studies on the anticancer effects of H_2_S donors^26–28^. H_2_S donors exhibit anti-cancer activities. NaHS is reported to have anti-cancer effects through the p38 Mitogen-activated protein kinase (MAPK) signalling pathway in C6 glioma cells^26^. GYY 4137 showed killing effects in seven human cancer cell lines and reduced tumour growth in a xenograft mouse model^22^. Garlic-derived H_2_S donor dialyl disulfide (DADS) enhanced the effect of eicosapentaenoic acid on cancer cell growth^29, 30^. However, little is known about the anti-cancer mechanism of H_2_S.

H_2_S acts as an inhibitor of PDE that boosts cyclic nucleotide and causes vasorelaxation *in vivo* ^31^. The present study aimed to determine the impact of H_2_S on the effect of anti-cancer agents. Our study assessed the impact of H_2_S on the anti-cancer effects of EGCG. We used NaHS as an H_2_S donor to minimize off-target effects because the mechanisms of NaHS-derived H_2_S release are very simple with few by-product compared with those of other H_2_S donors such as GYY 4137 and DATS.

In this study, we showed that the H_2_S donor NaHS synergically potentiated the anti-MM effect of EGCG through cGMP-PDE inhibition. Furthermore, other H_2_S donors GYY 4137 and DATS also significantly amplified the EGCG-elicited cGMP-dependent apoptosis-inducing signalling pathway. Combination treatment with EGCG and NaHS showed not only extended the survival period but also inhibited tumour growth in a xenograft mouse model. Collectively, our results suggest that the combination of H_2_S donor and EGCG maybe a useful approach for cancer-specific chemotherapy.

## Results

### H_2_S donors potentiate EGCG-induced cancer-specific cell death

**Figure 1:**
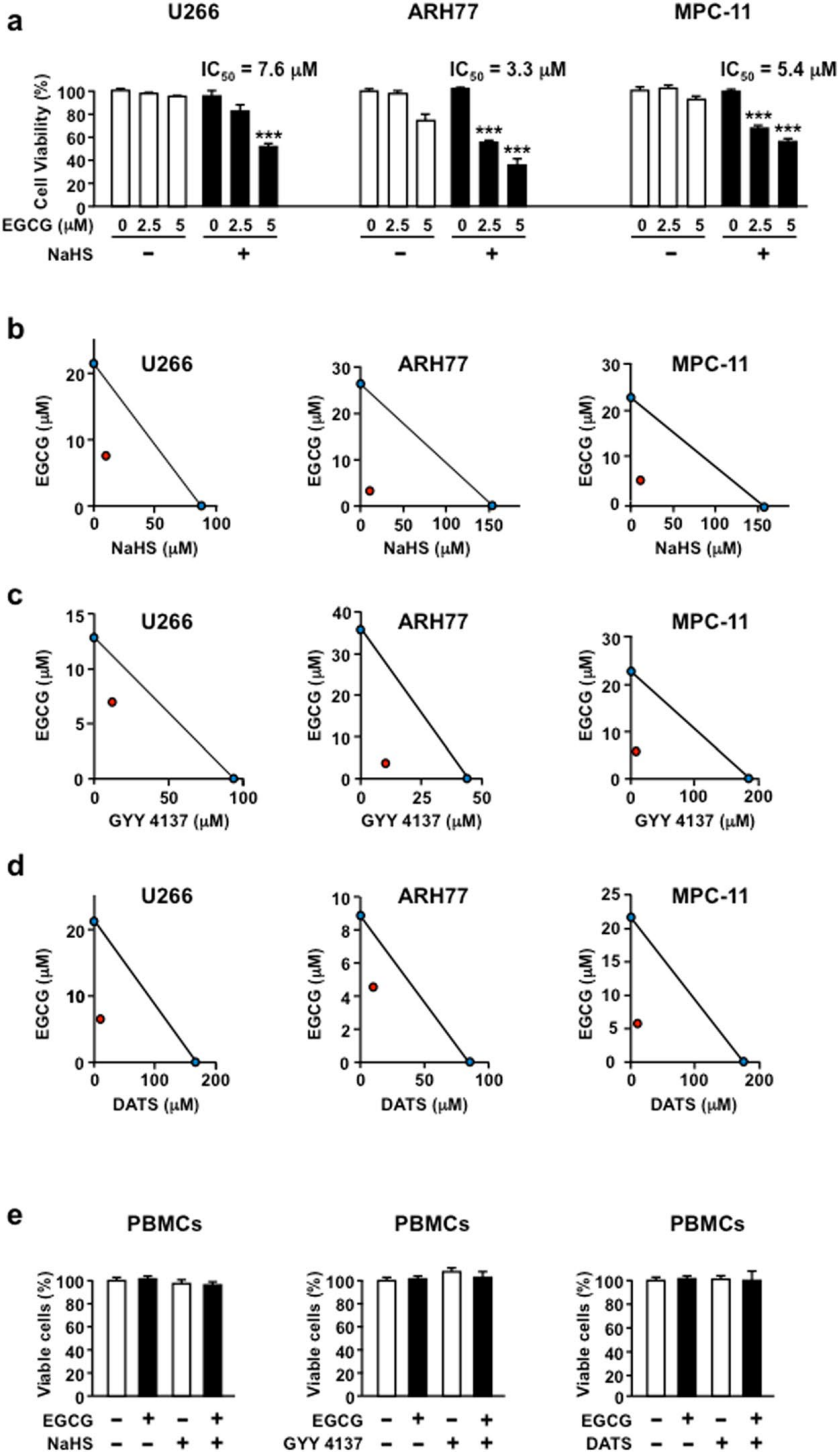
H_2_S donors potentiate EGCG cancer-specific cell death. (**a**) U266, ARH77 and MPC11 cells are cultured with or without NaHS (10 µM) and/or EGCG for 96 h, and viable cell numbers are measured. (**b**–**d**) The combination effect of EGCG and several H_2_S donors in U266, ARH77 and MPC-11 cells are measured by isobologram analysis. (**e**) Normal PBMCs are treated with NaHS, GYY ﻿4137 and DATS (10 µM) with or without EGCG (5 µM) for 96 h. Data are presented as mean ± SEM (*n* = 3). **P < 0.01, ***P < 0.001.

EGCG selectively kills cancer cells by targeting the overexpression of 67LR^2, 7, 8, 32^. However, the killing activity of EGCG at physiological concentration is limited^33^. We assessed the anti-cancer effect of the combination of EGCG and an H_2_S donor NaHS in the three MM cell lines. NaHS pretreatment significantly potentiated the anti-MM effect of EGCG, with 50% inhibitory concentrations (IC_50_) values of 7.6 µM (U266), 3.3 µM (ARH77) and 5.4 µM (MPC-11) in the cell lines, while the IC_50_ values for EGCG were 21.3 µM (U266), 25.8 µM (ARH77) and 21.6 µM (MPC-11) in the cell lines (Fig. 1a,b). H_2_S is a gasotransmitter that regulates blood pressure^34^ and penile erection^35^. However, little is known about its anti-cancer effect. The IC_50_ of NaHS was 88 µM (U266), 154.3 µM (ARH77) and 158 µM (MPC-11) in the cell lines (Supplementary Fig. 1). The isobologram plot is a well-used method for evaluating synergy based on the dose-response relationship of individual drugs. A straight line was noted on plotting the IC_50_ doses of EGCG and NaHS on the x- and y-axes, respectively. Isobologram analysis of growth-inhibition curves revealed that the combination of EGCG and NaHS was not simply additived but was synergistic in all the three cell lines (Fig. 1a,b). The same results were obtained for other H_2_S donors including GYY 4137 and DATS (Fig. 1c,d).

Selective toxicity is the most important factor in the anti-cancer effect. We found that all three H_2_S donors (NaHS, GYY 4137 and DATS) and EGCG showed a significant anti-MM effect without any negative effect to normal human peripheral blood mononuclear cells (PBMCs) (Fig. 1e). Collectively, the H_2_S donors synergistically potentiated the anti-MM effect of EGCG without any negative effect to normal cells.

### Combination of EGCG and NaHS induces apoptosis in MM cells

**Figure 2:**
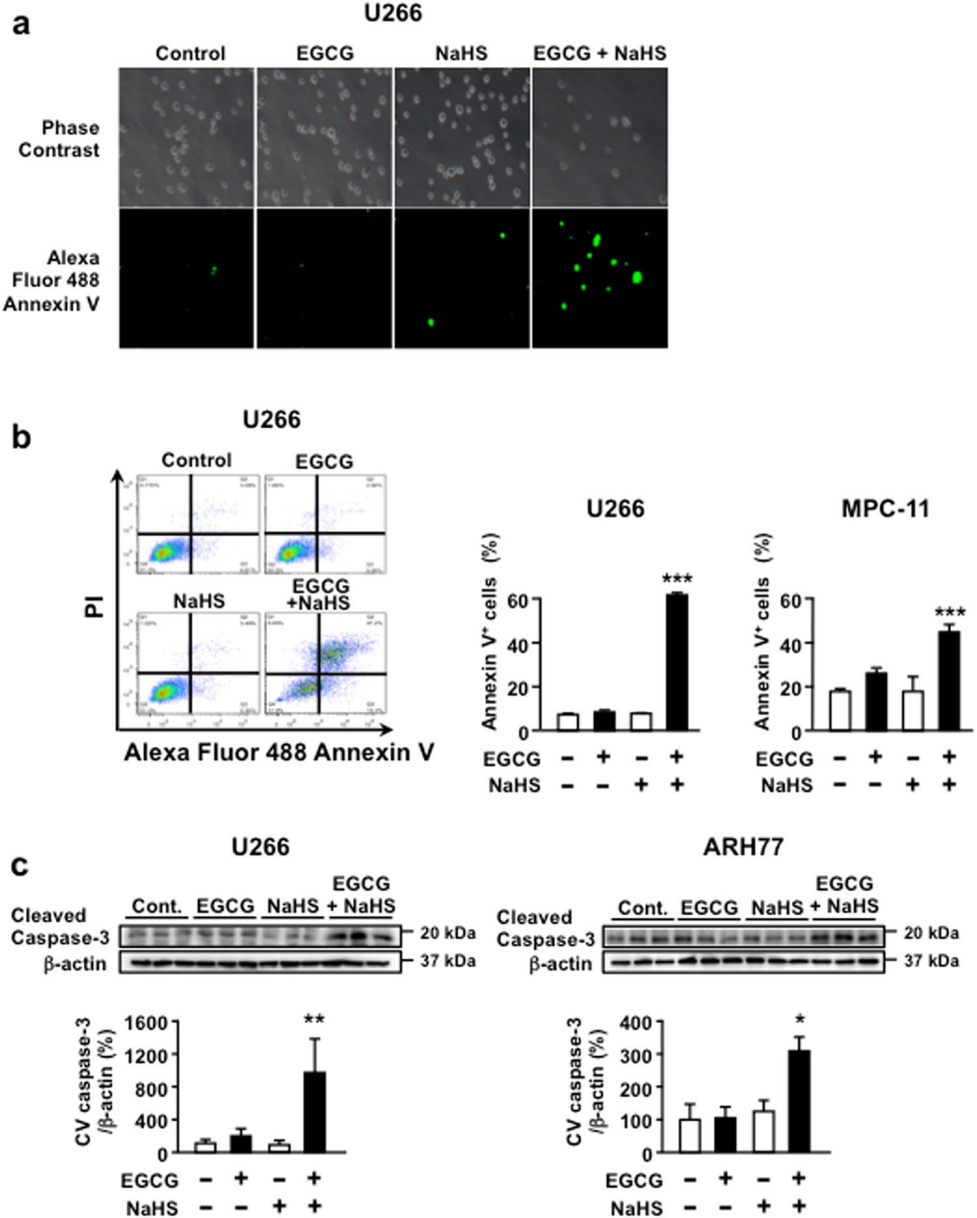
Combination of EGCG and NaHS induces apoptosis in MM cells. (**a**) U266 cells are treated or not treated with 5 μM EGCG in the presence or absence of 10 μM NaHS for 96 h and are observed under a florescence microscope. (**b**) Apoptotic cells are double stained with PI and Annexin V–Alexa Fluor 488 in U266 and MPC-11 cells. (**c**) U266 and ARH77 cells (human MM) are treated or not treated with 5 μM EGCG in the presence or absence of 10 μM NaHS for 72 h, and cleaved caspase-3 levels are assessed using western blot analysis. Cropped gels/blots are displayed. Full-length gels/blots are included in the Supplementary Information (Supplementary Fig. 7). Data are presented as mean ± SEM (*n* = 3). **P < 0.01, ***P < 0.001.

EGCG induces apoptotic cell death in MM cells^4, 7^. To investigate whether the combination of EGCG and NaHS induces apoptosis in MM cells, cells were treated with a combination of EGCG and NaHS and were stained with Annexin V–Alexa Fluor 488. These combination significantly potentiated apoptosis induction in the MM cell lines U266, ARH77 (human MM) and MPC-11 (mouse myeloma) cells (Fig. 2a,b). Furthermore, we found that the level of cleaved caspase-3, a key mediator in apoptosis, significantly increased in EGCG/NaHS-treated human MM cells (Fig. 2c). Taken together, these findings suggest that the H_2_S donor NaHS potentiates apoptosis-inducing activity of EGCG in MM cells.

### NaHS amplifies cGMP-dependent apoptosis signalling pathway

**Figure 3:**
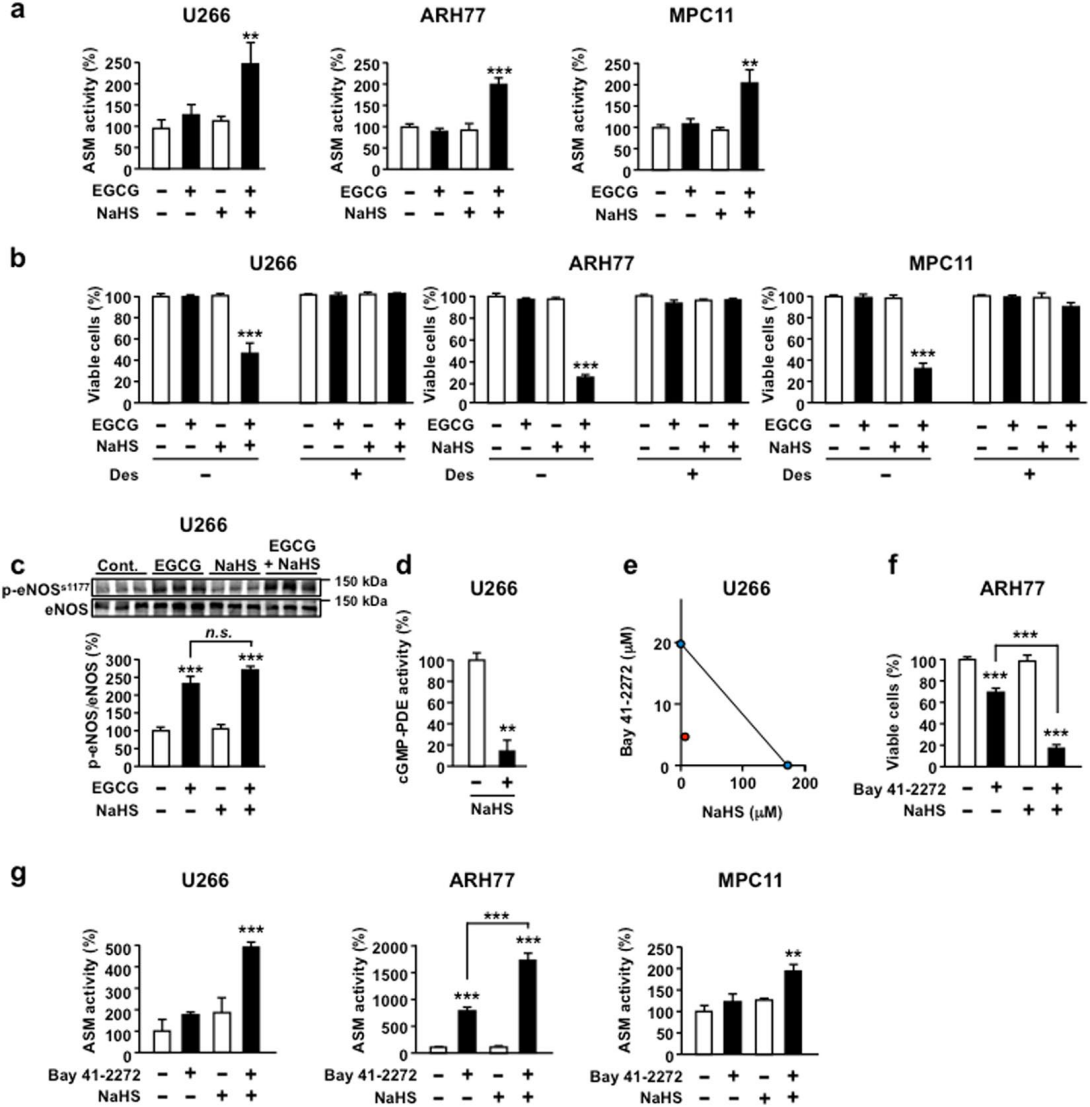
NaHS amplifies cGMP-dependent apoptosis signalling pathway. (**a**) The indicated cells are treated or not treated with 5 μM EGCG in the presence or absence of 10 μM NaHS and ASM activity is assessed using TLC analysis. (**b**) Multiple myeloma cell lines are pretreated or not pretreated with the ASM-specific inhibitor desipramine (Des; 5 μM) for 3 h and then are treated or not treated with EGCG (5 μM) and/or NaHS (10 µM) for 96 h. (**c**) U266 cells are treated or not treated with 5 μM EGCG in the presence or absence of 10 μM NaHS for 3 h, and phosphorylation of eNOS at Ser1177 is assessed using western blot analysis. Cropped gels/blots are displayed. Full-length gels/blots are included in the Supplementary Fig. 7. (**d**) Effect of 10 µM NaHS on cGMP-PDE enzyme activity in U266 cells. (**e**) Isobologram analysis of the effect of the combinations of Bay 41–2272 and NaHS. (**f**) ARH77 cells are treated or not treated with 5 μM Bay 41–2272 in the presence or absence of 10 μM NaHS for 96 h. (**g**) The indicated cells are treated or not treated with 5 μM Bay 41–2272 in the presence or absence of 10 μM NaHS, and ASM activity is assessed using TLC analysis. Data are presented as mean ± SEM (*n* = 3). **P < 0.01, ***P < 0.001.

Previously, we reported that EGCG initiated apoptosis by activating the cGMP/Protein Kinase C delta (PKCδ)/Acid sphingomyelinase (ASM) axis in MM cells^4, 32, 36^. To examine the role of the cGMP-dependent cell death pathway in EGCG and NaHS combination-induced cell death, we evaluated the involvement of ASM, a downstream mediator of cGMP-elicited cell death, in MM cells. The combination of EGCG and NaHS upregulated ASM activity in MM cell lines U266, ARH77 and MPC-11 cells (Fig. 3a). In addition, the ASM inhibitor desipramine (Des) abrogated the anti-MM effect of the combination treatment of EGCG and NaHS in U266, ARH77 and MPC-11 cells (Fig. 3b). To determine the effect of NaHS on the upstream signal of cGMP, we evaluated the effect of NaHS on EGCG-elicited phosphorylation of endothelial NOS (eNOS) at Ser1177. EGCG elicited phosphorylation of eNOS at Ser1177, but NaHS did not affect the EGCG-induced phosphorylation of eNOS at Ser1177 (Fig. 3c).

PDE is a well-established negative regulator of cGMP^17, 18^. Importantly, we found that NaHS reduced the enzyme activity of cGMP-PDE in U266 cells (Fig. 3d), although it did not affect the protein level of PDE5A (Supplementary Fig. 2).

Moreover, the combination of EGCG and NaHS increased cGMP levels in MM cells (Supplementary Fig. 3).

H_2_S can be endogenously produced in mammalian cells. We assessed the effect of EGCG on H_2_S production and cystathionine γ-lyase (CSE) expression, the major enzyme involved in H_2_S production^37^. EGCG did not affect both H_2_S production and CSE expression in MM cells (Supplementary Figs 4 and 5).

NaHS synergically potentiated the anti-MM effect of the NO-independent cGMP inducer Bay 41–2272 (Fig. 3e,f). To determine the effect of an H_2_S donor on cGMP-dependent cell death signalling, we assessed the effect of NaHS on Bay 41–2272-induced ASM activation. NaHS potentiated Bay 41–2272-elicited ASM activation in all three MM cell lines (Fig. 3g).

Taken together, these results suggest that NaHS potentiated EGCG-induced cell death through enhancement of cGMP-dependent cell death signalling in MM cells.

### NaHS potentiates the anti-MM effect of EGCG *in vivo*

**Figure 4:**
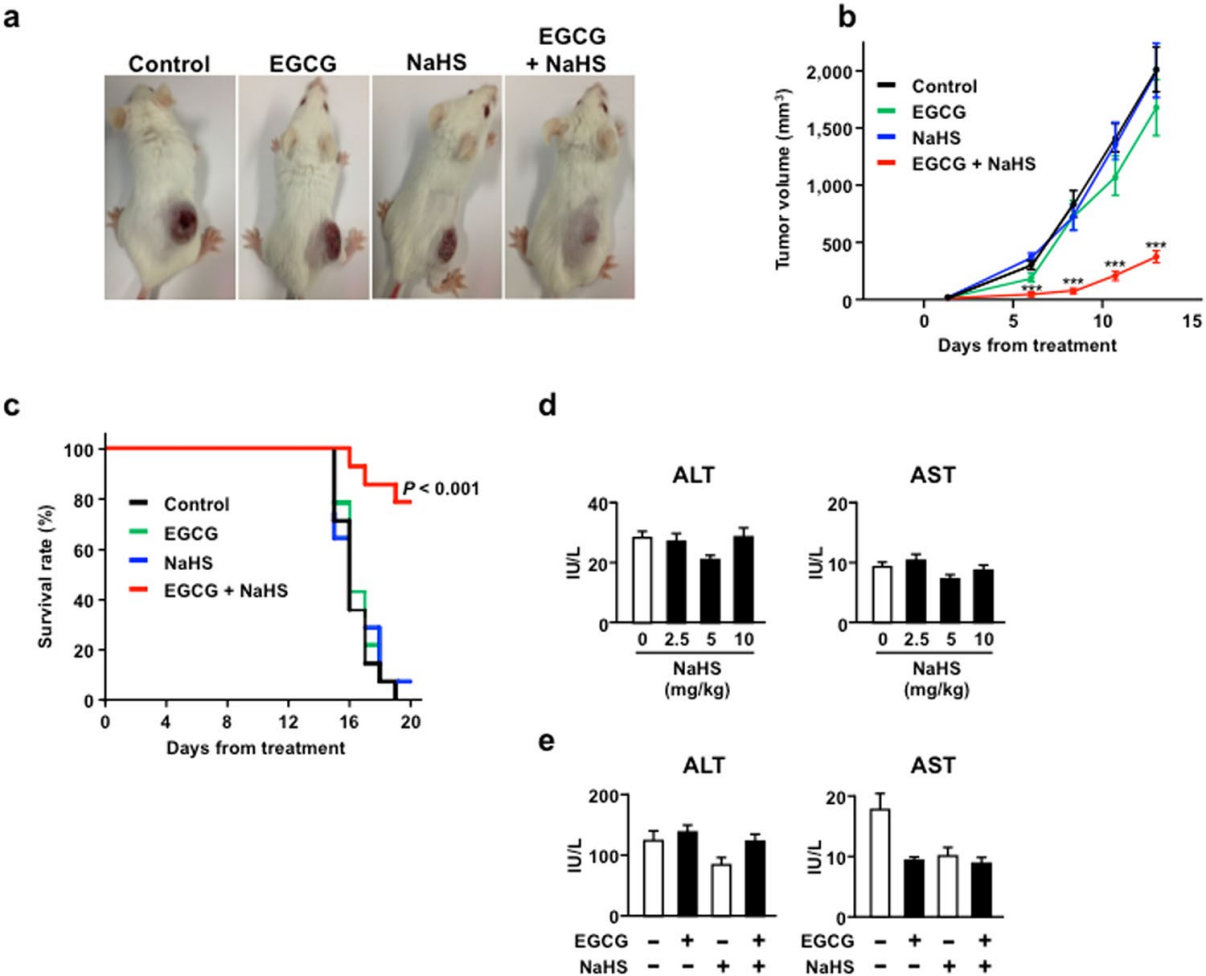
NaHS potentiates the anti-MM effect of EGCG *in vivo*. (**a**–**c**) MPC-11 cells are injected subcutaneously into female BALB/c mice, and mice (*n* = 14 per group) are administered i.p. injections of EGCG (15 mg/kg) and/or NaHS (10 mg/kg) every 2 days. Log-rank analyses of Kaplan–Meier curves are performed (*n* = 14). (**d**) Effect of the indicated NaHS concentration on the serum AST and ALT levels (*n* = 5). (**e**) Effect of the combination of EGCG and NaHS on the serum AST and ALT levels. Data are presented as mean ± SEM (*n* = 13). ***P < 0.001.

To evaluate the anti-cancer activity of the combination of EGCG and NaHS in a xenograft mouse model, MPC-11 cells were injected subcutaneously into 5-week-old BALB/c female mice. After the appearance of palpable tumours, the mice were administered intraperitoneal (i.p.) injections of EGCG (15 mg/kg/day) and/or NaHS (10 mg/kg/day) every 2 days. The combination of EGCG and NaHS resulted in significant suppression of tumour growth in the mouse model, while EGCG or NaHS alone did not show any effect on tumour growth (Fig. 4a,b). Moreover, log-rank analyses of the Kaplan–Meier survival curves showed significantly better survival among mice treated with the combination of EGCG and NaHS than among mice treated with PBS (control group), EGCG alone, or NaHS alone (Fig. 4c).

We evaluated the serum ALT/AST levels during NaHS treatment. BALB/c mice were administered intraperitoneal injections of NaHS (2.5 mg/kg/day, 5 mg/kg/day and 10 mg/kg/day) or PBS every 2 days. NaHS did not increase the serum ALT/AST levels when compared with the levels in the control group (Fig. 4d). In addition, there were no significant changes in mice tissue weights (heart, kidney, liver, spleen and lung) and mice body weights (Supplementary Fig. 6). Furthermore, the combination of EGCG and NaHS did not increase the serum ALT/AST levels (Fig. 4e).

### EGCG and NaHS amplify the tumour apoptosis in mouse xenograft model

**Figure 5:**
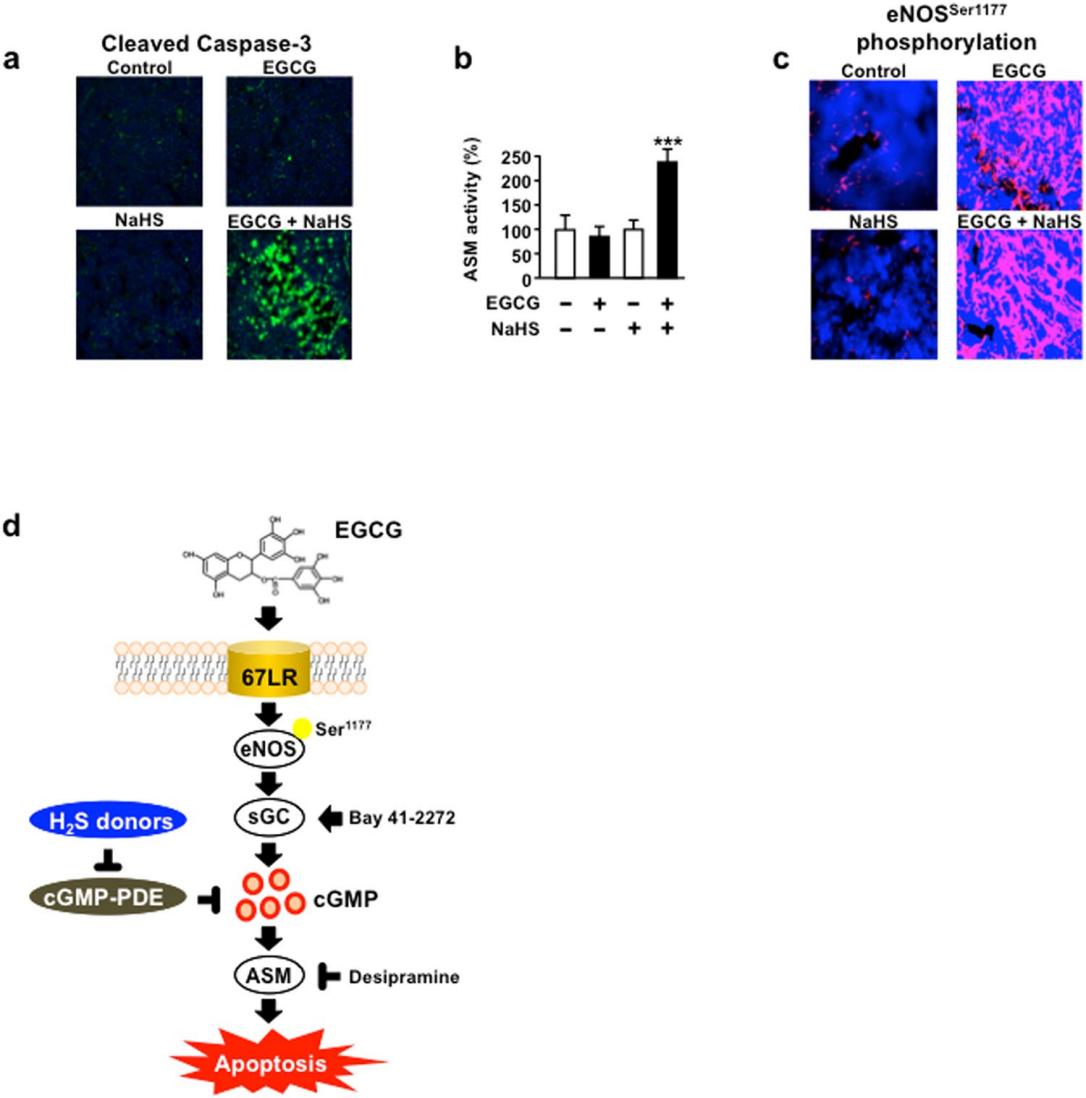
EGCG and NaHS amplify the tumour apoptosis in mouse xenograft model. (**a**) MPC-11 cells are injected subcutaneously into female BALB/c mice, and mice are administered i.p. injections of EGCG (15 mg/kg) and/or NaHS (10 mg/kg) every 2 days. Tumours are harvested and evaluated for cleaved caspase-3 (*n* = 6). (**b**) Tumours are harvested and evaluated for ASM activity (*n* = 13). (**c**) MPC11 cells are injected subcutaneously into female BALB/c mice, and mice are administered i.p. injections of EGCG (15 mg/kg) and/or NaHS (10 mg/kg) every 2 days. Tumours are harvested and evaluated for eNOS phosphorylation at Ser1177 (*n* = 6). (**d**) Schematic representation of the H_2_S donor potentiates EGCG-induced cell-death pathway. Data are presented as mean ± SEM. ***P < 0.001.

We confirmed the effect of the combination of EGCG and NaHS in a tumour- bearing model based on immunofluorescence analysis. Neither EGCG (15 mg/kg/day) nor NaHS (10 mg/kg/day) caused significant induction of cleaved caspase-3 levels in tumour tissues compared with that in control group. However, the combination of EGCG and NaHS (i.p.) increased cleaved caspase-3 levels (Fig. 5a). Moreover, the combination of EGCG and NaHS significantly upregulated the ASM activity (Fig. 5b). On the other hand, EGCG elicited phosphorylation of eNOS at Ser1177, which is an upstream signal of cGMP, but NaHS did not affect the phosphorylation of eNOS at Ser1177 (Fig. 5c). We showed schematic representation indicating that H_2_S donor potentiates EGCG-induced cell death pathway (Fig. 5d).

## Discussion

The gasotransmitter NO is produced by eNOS and regulates sGC for cGMP production^10–13^. The cGMP axis plays a key role in MM-specific cell death and mediates the MM-killing activity of EGCG^4^. However, the activity of EGCG at physiological concentrations is limited in MM cells^33^.

Here we showed that H_2_S donors, including NaHS, GYY 4137 and DATS, potentiate EGCG-induced cell death in MM cells. Moreover, the combination of EGCG and H_2_S donor extended the survival time and reduced the tumour volume in a xenograft mouse model.

Several studies have reported the growth-suppressing and apoptosis-inducing effects of H_2_S or H_2_S donors through the Bax, Blc-X and p38 MAPK signalling pathways^26–29^. In our study, three different H_2_S inducers strongly potentiated EGCG-induced cell death at very low concentrations (H_2_S donor 10 μM *in vitro*; NaHS, 10 mg/kg body weight *in vivo*) than those that are enough to induce anti-cancer effects (NaHS, approximately 200–1000 μM *in vitro*; GYY4131, 100–300 mg/kg/day). In this study, we observed that the H_2_S donor NaHS suppressed the enzyme activity of cGMP-PDE, the negative regulator of cGMP. Moreover, NaHS potentiated EGCG-induced cGMP production level.

Activation of the ASM axis is an essential mediator in cGMP-initiated apoptosis^4, 7^. Our data also showed that the ASM activity and cleaved caspase-3 levels increased in tumour tissues of mice treated with combination of EGCG and NaHS compared with those in control group. In contrast, compared with control group, EGCG or NaHS alone did not affect both ASM activity and cleaved caspase-3 levels. Furthermore, we confirmed that the H_2_S donor potentiated ASM activation induced by Bay 41–2272, a NO-independent sGC activator. The EGCG-induced eNOS/cGMP/ASM/caspase-3 axis plays an important role in MM cell death^4^. Single NaHS treatment does not appear to induce phosphorylation of eNOS at Ser1177 *in vitro* and *in vivo*. We also confirmed that NaHS did not have an impact on EGCG-elicited phosphorylation of eNOS at Ser1177. These results suggest that H_2_S donor potentiates the anti-MM effect of EGCG by enhancing the downstream of cGMP but not affecting eNOS phosphorylation at Ser1177. Moreover, H_2_S is known as an inhibitor of PDE5 activity^31^. Furthermore, EGCG did not affect H_2_S production in MM cells. These results suggest that intracellular H_2_S produced by NaHS potentiates EGCG-induced anti-MM effect via inhibition of cGMP-PDE enzyme activity.

We also showed that H_2_S donors dramatically potentiated the anti-cancer effects of EGCG at a physiological concentration without affecting normal PBMCs. With regard to efficient chemotherapy, novel therapeutic drugs with different mechanisms and highly selective toxicity are required. Hepatotoxicity is a well-known adverse effect of high-dose EGCG^38^ and, in some cases, elevation of the transaminases ALT and AST has been observed in clinical trials of EGCG^39^. Importantly, the combination of EGCG and NaHS did not increase the serum ALT/AST levels, suggesting that an H_2_S donor could be a potent candidate to enhance the pharmacological effect of EGCG without enhancing its adverse effects.

In conclusion, H_2_S donor potentiates the anti-MM effect of EGCG at a physiological concentration. The combination of EGCG and an H_2_S donor could provide a novel strategy for MM treatment without affecting normal PBMCs. Moreover, our data suggest that H_2_S may potentiate the downstream mediators of EGCG-induced apoptosis along with the inhibition of cGMP-PDE enzyme activity. PDE5 overexpressed in many types of cancer cells, such as a chronic lymphocytic leukaemia, acute myeloid leukaemia, stomach cancer, pancreatic cancer, prostate cancer, breast cancer and MM cells^4, 5, 32^. EGCG and PDE5 inhibitor in combination also induce apoptotic cell death in these cancer cells^4, 5, 32^. Taken together, we suggest that H_2_S donor and EGCG in combination could be effective for chemotherapy and have high potential for use in various types of cancers including MM.

## Materials and Methods

### Cell culture

Primary PBMCs were provided by Takara (Siga, Japan). U266, ARH77 (human MM) and MPC-11 (mouse myeloma) cell lines were cultured in RPMI 1640 supplemented with 10% (v/v) foetal bovine serum (FBS), 100 U/ml penicillin and 100 µg/ml streptomycin at 37 °C in 5% CO_2_ and 100% humidity.

MM cells were seeded into 24-well plates at a seeding density of 5 × 10^4^ cells/well and were then treated with drugs at various concentrations or indicated periods in RPMI 1640 medium supplemented with 1% FBS, 200 U/ml catalase and 5 U/ml superoxide dismutase (SOD) (Sigma-Aldrich).

Cell viability was evaluated using trypan-blue exclusion. Apoptotic cells were detected by using the Alexa Fluor 488 Annexin V apoptosis detection kit (Invitrogen). Cells were suspended in Annexin V–Alexa Fluor 488 and Annexin V binding buffer. A portion of the cell suspension was then placed onto a glass slide. All images of *in vitro* experiments were acquired using a fluorescence microscope (BZ-8100; Keyence). For flow cytometric analyses, the percentages of Annexin-V^+^ cells were calculated by combining Annexin-V^+^/PI^-^ (early apoptosis) and Annexin-V^+^/PI^+^ (late apoptosis) cells, followed by analyses on a Verse^TM^ system (Becton Dickinson). Measurement of ASM activity was performed as described previously^4^.

Evaluation of intracellular cGMP was performed by using the cGMP assay kit (Cayman Chemicals), according to the manufacturer’s protocol. Analysis was carried out by using the Envision™ Plate Reader (Perkin–Elmer).

### Immunoblot analysis

Cells were lysed in a lysis buffer containing 50 mM Tris-HCl (pH 7.5), 150 mM NaCl, 1% Triton X-100, 1 mM EDTA, 50 mM NaF, 30 mM Na_4_P_2_O_7_, 1 mM phenylmethanesulfonyl fluoride, 2 mg/ml aprotinin and 1 mM pervanadate.

Approximately 50 μg protein was suspended in Laemmli sample buffer (0.1 M Tris-HCl buffer, pH 6.8; 1% SDS; 0.05% mercaptoethanol; 10% glycerol and 0.001% bromophenol blue), boiled, and electrophoresed on SDS-polyacrylamide gels. SDS gels were then electroblotted onto Trans-Blot nitrocellulose membranes (Bio-Rad). Blots were incubated with the indicated antibodies in Tween 20-PBS (TPBS) containing 1% BSA. The blots were then washed with TPBS and incubated with anti-rabbit or anti-mouse HRP conjugates. After washing, specific proteins were detected using an enhanced chemiluminescence system according to a manual from Amersham Life Sciences.

### Xenograft murine model

Five-week-old female BALB/c mice were obtained from Kyudo (Fukuoka, Japan). Mice were inoculated subcutaneously in the interscapular area with 1 × 10^6^ MPC-11 cells in 200 µl of RPMI 1640 medium. Following the appearance of palpable tumours, the mice were injected with saline alone, or EGCG (15 mg/kg) or NaHS (10 mg/kg) every 2 days. The tumour sizes were evaluated by using callipers, and their volumes were calculated as (length) × (width)^2^ × 0.5. All animal studies were performed in accordance with the law (protocol no. 105) and notification (protocol no. 6) of the Japanese government for the welfare of experimental animals. The study protocol was approved by the Animal Care Committee.

Immunofluorescent staining experiments were performed by using a fluorescence microscope (BZ-9000; Keyence). Briefly, anti-cleaved caspase-3 antibodies (D175) (Cell Signalling Technology) were used at 1:300 dilution. Slides were then treated with Alexa Fluor 488–conjugated secondary antibody (Invitrogen) at 1:300 dilution and incubated for 1 hour. To evaluate the effect of NaHS, mice were injected with saline alone or indicated NaHS (i.p.) every 2 days for 8 weeks. At the end of 8 weeks of treatment, the mice were anaesthetized under isoflurane vapour after overnight food deprivation and each organ were weighted and the serum ALT and AST levels were evaluated. The AST and ALT quantification kits were purchased from Wako (Osaka, Japan).

### Statistical analysis

All data are expressed as mean ± SEM. The IC_50_ value calculation and isobologram analysis were performed by using the Calcusyn 2.0 software (Biosoft). The significance of differences between experimental variables was determined using Tukey’s test. Statistical analyses were performed using the KyPlot software (Kyens Lab, Tokyo, Japan). Survival was assessed using the log-rank test for Kaplan–Meier curves. A *P*-value < 0.05 was considered significant.

## Electronic supplementary material

Supplementary information
