# Supplementary material for: Hydrogen sulphide donors selectively potentiate a green tea polyphenol EGCG-induced apoptosis of multiple myeloma cells
Source: Sci Rep. 2017 Jul 27;7:6665. doi: 10.1038/s41598-017-06879-5 (PMC5532223; doi:10.1038/s41598-017-06879-5)
Supplement: Supplementary file 1 — Supplementary information [file 41598_2017_6879_MOESM1_ESM.pdf]

Hydrogen sulphide donors selectively potentiate a green tea polyphenol EGCG-induced apoptosis of multiple myeloma cells

Jaehoon Bae, Motofumi Kumazoe, Shuya Yamashita & Hirofumi Tachibana

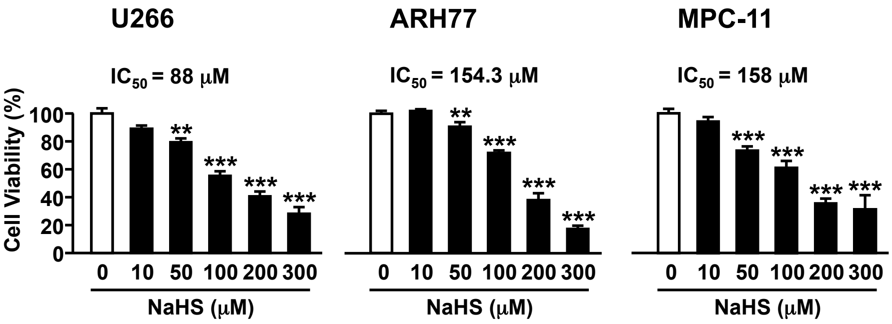

Supplementary Fig. 1: Cell death-inducing activity of NaHS in MM cells.
U266, ARH77 and MPC11 cells are treated with NaHS for 96 h at the indicated concentrations, and viable cell numbers are measured. The IC_{50} value calculations are performed. Data are presented as mean ± SEM (*n* = 3). $^{**}P < 0.01$, $^{***}P < 0.001$.

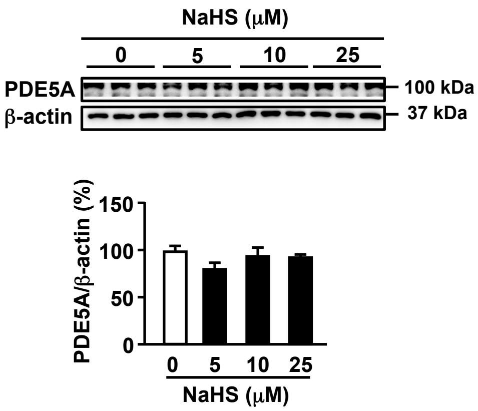

Supplementary Fig. 2: NaHS has no effect on the protein level of PDE5A.
U266 cells are treated with NaHS for 3 h at the indicated concentrations, and protein level of PDE5A is assessed by western blot analysis. Data are presented as mean ± SEM (*n* = 3).

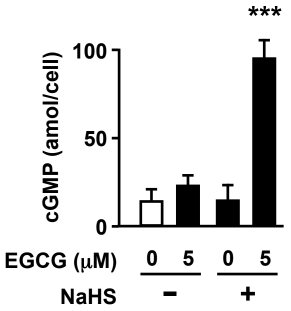

Supplementary Fig. 3: NaHS potentiates cGMP-inducing effect of EGCG.
U266 cells are treated with 10 μM NaHS and/or indicated concentrations of EGCG for 3 h, and cGMP levels is asseesed. Data are presented as mean ± SEM (n = 3). ***$P < 0.001$.

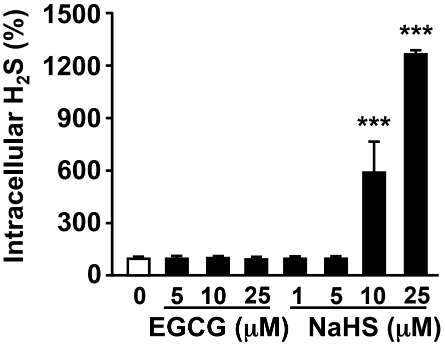

Supplementary Fig. 4: The effect of EGCG or NaHS on intracellular H_2S production.
U266 cells are treated with EGCG or NaHS for 3 hours at the indicated concentrations, and intracellular H_2S are measured by highly selective fluorescence probe HSip-1 (λex 491 nm, λem 516 nm). Data are presented as mean ± SEM (*n* = 3). ***$P < 0.001$.

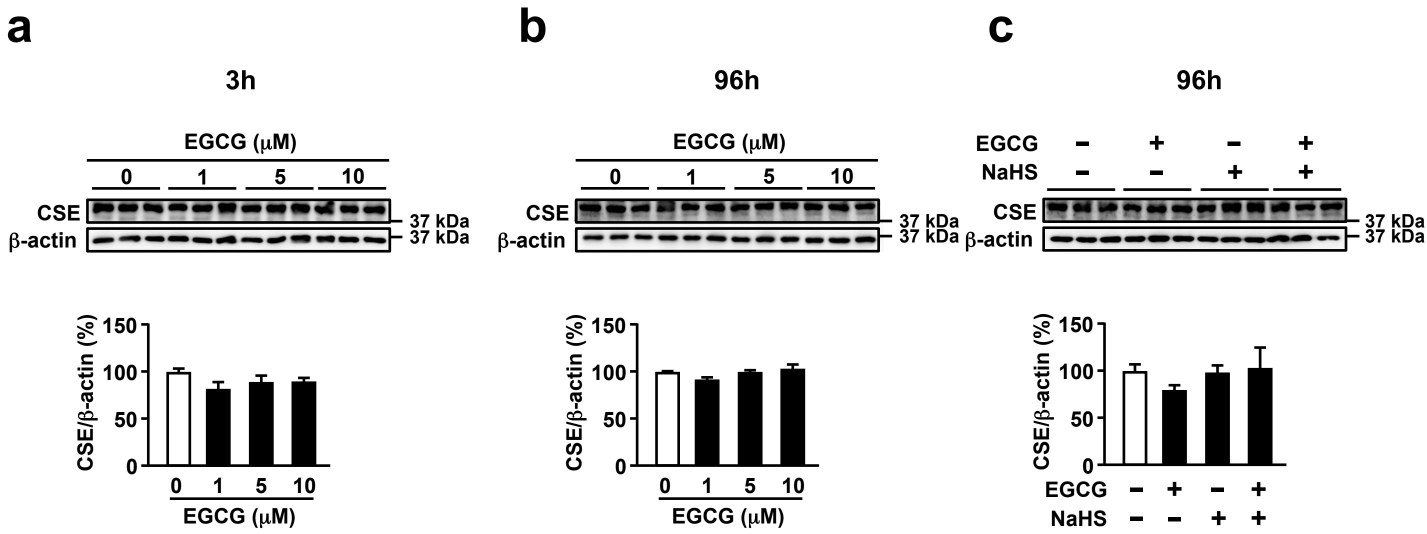

Supplementary Fig. 5: EGCG and/or NaHS have no effect on CSE expression.
(a) U266 cells are treated with EGCG for 3 h at the indicated concentrations, and protein level of CSE is assessed by western blot analysis. (b) U266 cells are treated with EGCG for 96 h at the indicated concentrations, and protein level of CSE is assessed by western blot analysis. (c) U266 cells are treated with 5 μM EGCG and/or 10 μM NaHS for 96 h, and protein level of CSE is assessed by western blot analysis. Data are presented as mean ± SEM (*n* = 3).

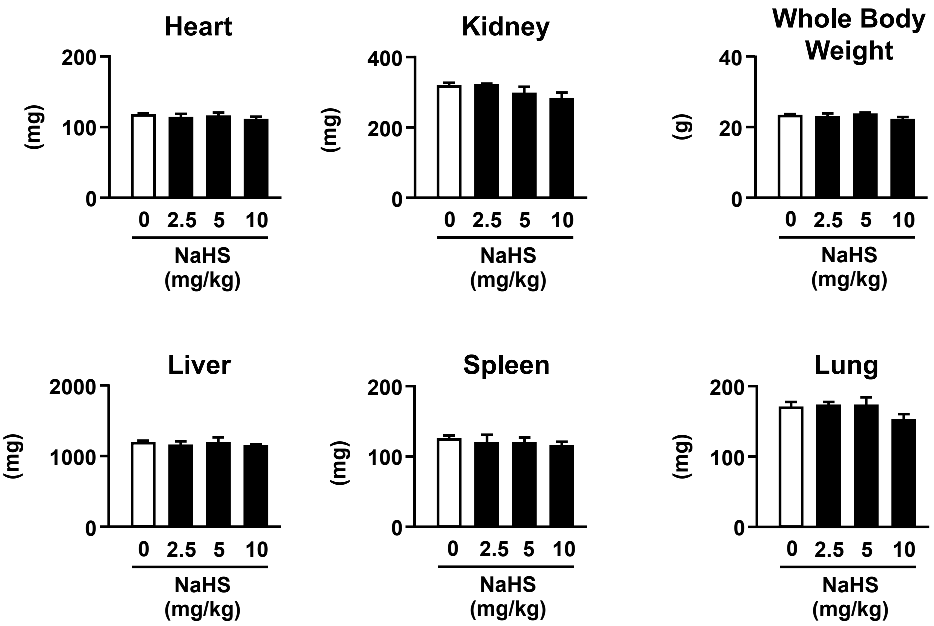

Supplementary Fig. 6: Evaluation on the toxicity of NaHS *in vivo.*
Five-week-old BALB/c mice (n = 14 per group) are administered i.p. injections of NaHS every 2 days at the indicated concentrations. Heart, kidney, liver, spleen and lung tissue weights and mice body weights are evaluated after 8 weeks. Data are presented as mean ± SEM.

Fig 2c

U266

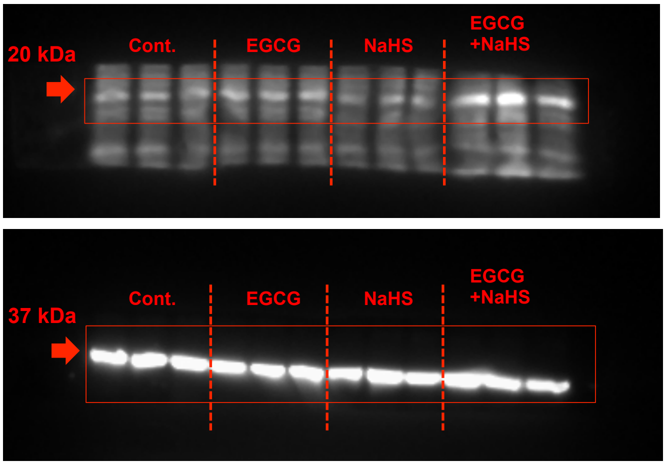

Fig 2c

ARH77

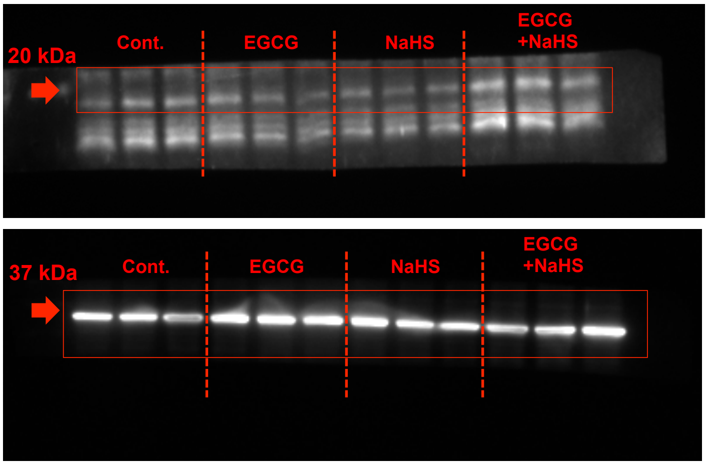

Fig 3c

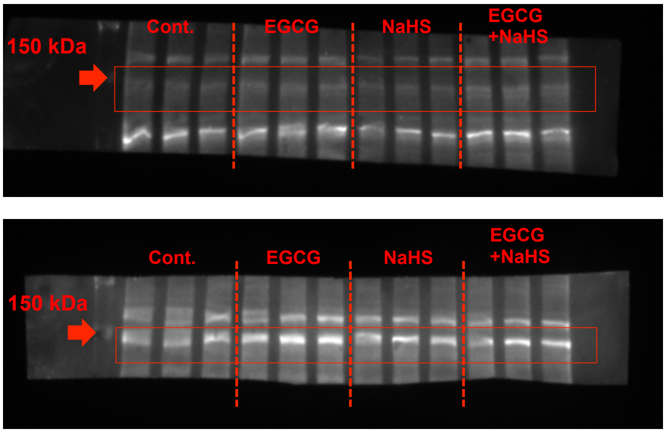

Supplementary Fig. 7: Uncut blot data of Figure2c and Figure 3c.
